# “I think the humanity just gets lost over and over again”: A phenomenological study of the experiences of higher-weight medical students

**DOI:** 10.1371/journal.pone.0340598

**Published:** 2026-01-08

**Authors:** Sebastian C. K. Shaw, Laura R. Hennessy-Grainger, Angela Meadows

**Affiliations:** 1 Department of Medical Education, Brighton and Sussex Medical School, Brighton, East Sussex, United Kingdom; 2 Tavistock and Portman NHS Foundation Trust, London, United Kingdom; 3 Department of Psychology, University of Essex, Colchester, Essex, United Kingdom; Health Researcher, SPAIN

## Abstract

A growing body of research has found weight stigma to independently drive both morbidity and mortality, regardless of actual weight. This has, however, yet to translate into medical education and practice. Studies have shown doctors to be common sources of weight stigma, which may be driven, in part, by their medical training. Higher-weight doctors may be best placed to understand and support the health needs of higher-weight people. However, significant levels of implicit anti-fat bias towards higher-weight colleagues lingers in the medical profession. Inclusive practices and more holistic education around weight are therefore needed to support and retain higher weight doctors within the workforce, starting within medical schools. This may improve both staff experiences and patient care. This study aims to explore the experiences of higher weight medical students in the UK. This is an interpretive phenomenological study. Three higher-weight medical students (two women, 1 man, all 2^nd^ year medical students, BMI range 31–50 kg/m^2^) underwent loosely structured interviews over Microsoft Teams. These were audio-recorded. Audio recordings were transcribed verbatim and underwent an interpretive phenomenological analysis. Participants reported logistic and environmental issues, such a lack of provision of larger uniform sizes or narrow small lecture room seat sizes. They also reported negative experiences with peers, teachers, and the general public in relation to their size. Despite this, higher-weight doctors were indeed felt to be important to advocate for higher-weight patients. Negative experiences seemed to stem from wider sociocultural issues and reflect the intersectional nature of weight stigma. To improve matters in the longer term, medical schools should review and update their weight-related teaching, alongside considering the accessibility of their physical environments. Medical schools could consider weight stigma as part of their current efforts to decolonise medical curricula.

## Introduction

In recent years, providers of medical education have become more intentional about efforts to promote greater inclusivity in order to diversify the medical workforce to better represent the populations it serves [1]. This ethos is supported by both the General Medical Council (GMC) and the Medical Schools Council in the United Kingdom (UK) [1,2]. True inclusion, however, encompasses far more than mere recruitment, requiring equitable experiences within the profession. In the UK, the Equality Act (2010) prohibits discrimination on the grounds of nine ‘protected characteristics’: age, sex, race, disability, sexual orientation, gender reassignment, marital status, pregnancy, and religion [3]. Yet, despite efforts to promote diversity in the medical workforce, evidence suggests that we are yet to achieve our aim of true inclusion. For example, discrimination is still consistently reported by medical students and doctors across a range of protected characteristics [4–8]. Whilst legislated protected characteristics provide a starting point and benchmark against which to measure efforts, inclusivity should not be limited to these characteristics if we wish to create a workforce that is truly representative of the populations we serve – and thus better placed to meet their needs. Higher-weight status is one such example of a characteristic not formally covered by the Equality Act (2010) – and one that is strongly stigmatised in both wider society and the healthcare system [9]. Weight stigma has been defined as the “social devaluation, denigration, and marginalization” of higher-weight people [10]. Despite its lack of legal protections, discrimination on the basis of weight rivals the prevalence of both age and racial discrimination [11]. This is hardly surprising when viewed in the context of the wider “moral panic” around ‘obesity’ in wider society, whereby higher-weight people are stereotyped as “lazy”, “weak-willed”, and “unintelligent”, among other negative characteristics, whose health concerns are self-inflicted, and are thus deserving of disapproval and contempt [12,13]. This scapegoating allows for “the relinquishing of Government responsibility to appropriately address environmental and societal drivers” of health [14].

A substantial body of evidence now indicates that weight *per* se may not be a reliable marker of individual health, that body weight is largely resistant to manipulation efforts, at least in the long term, and that bodies can be metabolically healthy and unhealthy at all sizes, despite weight remaining a focus of healthcare and public health [10,15–20]. Furthermore, anti-fat attitudes have been demonstrated in a wide variety of healthcare professionals, including general practitioners and providers specialising in the care of higher-weight patients [21]. Such attitudes are linked to poorer doctor-patient relationships and may impede the delivery of safe, effective, and evidence-based healthcare [9,22,23]. Unsurprisingly, higher-weight people report stigma among healthcare professionals as a barrier to healthcare [24–26], resulting in future healthcare avoidance [27,28]. This in turn leads to higher-weight people presenting later to healthcare services with more severe conditions and increased likelihood of poor outcomes [29,30]. Considering the UK National Health Service core values of “respect and dignity” and “everyone counts” [31], there is clearly a disconnect here in need of addressing. As such, increased higher-weight representation within the medical workforce itself is an important consideration, echoing the GMC sentiment that “a diverse population is better served by a diverse workforce that has had similar experiences and understands their needs” [32]. While weight is not among the characteristics tracked in medical school equality and diversity statistics [21], baseline data from the US Medical Student Cognitive Habits and Growth Evaluation Study (CHANGES) cohort indicated that only 24.5% of 4687 matriculating students at 49 US medical schools had self-reported height and weight that would put them in the “overweight” or “obese” BMI categories [33] – considerably lower than U.S. population norms [34]. It is unclear whether this disparity is due to self-selection out of medicine or to structural barriers against acceptance. While both may play a role, it is well documented that higher-weight individuals face barriers to entry into tertiary and graduate education [35–39]

A growing body of research has shown weight stigma to be an independent driver for morbidity and mortality, even when controlling for actual weight or body mass index (BMI) [40,41]. For example, perceived weight discrimination doubles the relative risk of exhibiting high allostatic load – an indicator of metabolic dysregulation – over a 10-year period [42]. Consequently, weight stigma is also associated with elevated risk of a variety of chronic illnesses, including type 2 diabetes mellitus and cardiovascular disease, even after controlling for BMI, physical activity, and sociodemographic variables [43]. As such, the concept of weight stigma has started to gain traction in clinical academia in recent years. It has yet, however, to be explicitly reflected in medical training and practice [44]. Research has in fact shown that doctors are key sources of the weight stigma experienced by higher-weight people [9,45]. Furthermore, doctors have been found to harbour both implicit and explicit anti-fat biases against their higher-weight colleagues [46,47]. Such practices may call matters of professionalism into question [48].

Several small studies have explored medical students’ experiences of observed weight stigma during their training [49–52], although these are largely limited to cohorts at individual medical schools and tend not to consider the prevalence of weight stigma directed towards students, or the impact of weight stigma on the health, wellbeing, or educational experience of medical students. However, longitudinal data from the CHANGES study, which surveyed nearly 4,000 medical students from 49 US medical schools at the start of their first year of medical school and at the end of their fourth year, confirms weight stigma during medical training as a more systemic issue [21]. Almost 90% of students reported witnessing weight stigma directed toward patients, 50% reported discriminatory treatment of higher-weight patients, and just under a quarter had observed negative weight-related treatment directed at higher-weight medical students, including unfair evaluations, offensive remarks, and public humiliation [21]. In all cases, weight-related stigma was noticed more by heavier students, which may speak to how ingrained such practices are that they may not be recognised or recalled as readily by students outside the target group [21]. Observed weight stigma was associated with worse psychological and general health and increased medical school burnout across the cohort, but this was particularly pronounced in higher-weight students [21]. The effect was mediated, in part, by higher-weight, but not normative-weight, students feeling a reduced sense of belonging in medical school [21].

The present study forms part of series of work on weight experiences and weight stigma in medical education. This current study aims to explore the experiences of higher-weight medical students – and is the first to consider the impact of being the *target* of weight stigma on medical students themselves.

### Research team positionality

SS is an Associate Professor in Medical Education at Brighton and Sussex Medical School (BSMS). He has significant experience with phenomenological research [53–56] and has published guidance on its use in medical education [57]. He is also a doctor working in the UK National Health Service. His interest in this area stems from his own higher-weight status.

LH is a trainee in gender identity, following completion of the UK foundation programme in the NHS. She has some experience with phenomenological research [56]. Her interest here also stemmed from her own higher-weight status.

AM is a Lecturer in Psychology at the University of Essex. Her research focuses on individual and structural weight stigma. She has published on anti-fat attitudes and experiences of weight stigma in medical students [21,58], and on how medicalised terminology around body size perpetuates stigma in research and clinical care [59]. She identifies as fat.

## Methods

### Methodology

This is an interpretive phenomenological study. Phenomenology stems from philosophy and, as a research approach, refers to the qualitative study of lived experience [60]. Descriptive phenomenology traditionally required researchers to bracket out their own preconceptions and experiences in the design, conduct, and write up processes [57]. Interpretive phenomenology, however, considers matters more subjectively, and asserts that there are multiple realities for multiple people, which are best accessed through acknowledging, embracing, and including the subjective reality of the researcher [57]. Unlike descriptive phenomenology, interpretive phenomenology also concerns itself with how participants make sense of their own experiences, as interpreted by the researcher(s) [57]. This process of intersubjectivity, as a form of hermeneutic cycle [61], has been referred to as a “fusion of horizons” [62] and embodies the idiographic focus that is so important to interpretive phenomenological studies [61]. In essence, this approach embraces subjectivity and requires researchers to use their own insights to “read between the lines” in the search for deeper meaning. As such, interpretive phenomenological studies are well suited to insider research teams like our own.

### Ethical considerations

This study was approved by the BSMS Research Governance and Ethics Committee (reference: ER/BSMS9DB3/4). However, formal approvals are but one aspect of ethical qualitative research. Given the focus of this particular study, relational ethical aspects also needed to be considered [63]. For example, a medical educationalist doctor interviewing medical students risks an undeniable power dynamic. To navigate this, no participants were prior known to the research team. Furthermore, we gave extensive consideration to our use of language within the study, opting eventually for “higher-weight” in an effort to embody non-maleficence in our approach [59]. Our reasoning for this was also made clear to participants in advance of their interviews, grounded in openness about our insider status. For example, our participant information sheet stated the following:

*“We know that different people prefer to describe their weight in different ways. Our wording is not intended to cause offence, and does not convey any judgement on the part of the researchers, who are in ourselves of higher-weight status.”*

Given the potentially stigmatising nature of the topic under study, there was the possibility that participants might report experiences of a safeguarding nature. As such, we also had to consider reporting processes and confidentiality in depth as part of our planning. This was, again, made explicit to participants in advance of us requesting informed consent:

*“If you were to disclose experiences of abuse, we would ask your permission to report such issues. We will not do so without your permission. If you agree to us reporting such issues, we would not include your name. It may, however, be possible for the perpetrator(s) to recognise you from the experience. Whilst it is highly unlikely, if you were to disclose something that directly jeopardises the safety of yourself or others, the research team may be required to report this without your permission.”*

### Recruitment and sampling

A purposive sampling approach was used. Participants needed to be current medical students who self-identified as being higher-weight – they were asked to have a body mass index (BMI) over 25 kg/m^2^, equivalent to a BMI in the “overweight” category, or above. A brief study advertisement was emailed to all medical students within a single UK medical school. The advertisement outlined the study and invited anyone interested in participating to contact LH via email for further information. Those who made contact were sent a participant information sheet and a consent form to review – and were invited to schedule an interview with LH if they wished to proceed.

All those who expressed interest and met the inclusion criteria were accepted into this study. It is worth noting that recruitment took place during a COVID-19 lockdown, which undoubtedly impacted our final number of participants, due to the many other stresses impacting medical training at that time.

### Data collection

LH conducted one-to-one, online interviews with participants over Microsoft Teams, which were audio-recorded. These took place in March 2021 and ranged from 32 to 60 minutes in length. Informed consent was received verbally (separately audio-recorded) at the start of each interview. Interviews were loosely structured, exploring experiences in relation to:

- Choosing to study medicine/ medical school admissions
- Peers
- University teachers/staff
- Clinical supervisors/staff
- The public, related to medical training (e.g., patients)
- Any systemic issues relating to higher-weight status.

We chose to use loosely structured interviews to allow participants to best tell their own stories, without the constraints of specifically pre-worded questions (such as those used within semi-structured interviews). Instead, the aforementioned topic areas were used to guide the flow of the interviews, in an order that best suited the evolving conversation with each participant. These topic areas were iteratively constructed, grounded in the insider experiences of SS and LH.

### Data analysis

LH transcribed the interview audio-recordings verbatim. SS and LH then performed an interpretive phenomenological analysis using the approach of Smith et al [61]. SS and LH both immersed themselves in the transcripts through repeated reading. This was followed by exploratory noting, which was conducted using the Microsoft Word comments feature. Stepping away from the original data, the exploratory notes were then reviewed and condensed, to construct experiential statements. These stages were led by LH under SS’s guidance. Following this, SS explored the experiential statements to assess for potential connections. He then used these to construct personal experiential themes for each participant. SS then searched for patterns across the personal experiential themes and used these to construct our final, group experiential themes (GETs). Finally, SS and LH reviewed the overall analysis, cross-referencing with the original data sources. Both agreed on the final interpretation.

## Results

Three individuals participated (Table 1). None dropped out. None had any background relationships/connections with the research team. Each has been given a pseudonym.

**Table 1:** Participant information.

Pseudonym	Age	Gender	Year of medical school	Self-reported weight status
Carmen	22	Woman	2^nd^ year	BMI = 50.2 kg/m^2^
Steve	42	Man	2^nd^ year	“Morbidly obese”
Hannah	37	Woman	2^nd^ year	BMI = 31 kg/m^2^ (previously 36 kg/m^2^)

**Fig 1:**
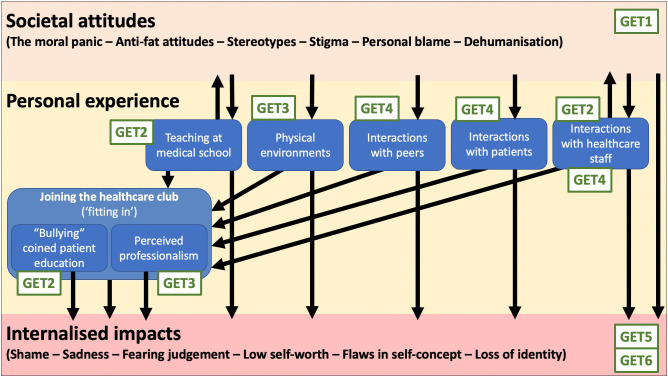
Visual representation of the interconnecting experiences of our participants. GET = group experiential theme.

Participants’ higher-weight status had pervasive impacts across most aspects of their lives, including their world views. Of note, there was an interesting discordance in the ways participants framed their specific experiences at a surface level. Carmen’s recollections largely related to the physical impacts of her weight on her day-to-day life. Steve, on the other hand, told a story of self-acceptance and the impact of higher-weight status on his own perspectives. Finally, Hannah framed her experiences in the context of extensive reading and insight into wider sociocultural issues. Our analysis constructed six GETs, which will be explored throughout the remainder of this section. Fig 1 explores the interconnection between the broad experiences discussed, mapped to our GETs.

### GET 1: Society and stereotypes – Is it me, or is it the world?

#### Thematic mapping.

This theme considers the broader external influences at play, which were felt to feed into the milieux and experiences of our participants on a more individual level.

Throughout the interviews, it became clear that all three participants had some awareness of a societal dislike for higher-weight people – and that all three had reported negative experiences throughout their lives.

*“You have barriers because our society is not very nice to us.”* (Hannah)

*“Even as a kid, you know kids would say, “you’re fat*.”” (Steve)

*“*[A stranger on a bus] *just immediately assumed that because I’m overweight, I cannot like bend down and tie my shoes.”* (Carmen)

All felt there was an expectation for them to fulfil certain stereotypes as higher-weight people, such as being lazy or unhealthy. As Carmen put it, *“people always associate obesity with laziness.”* Steve also spoke to this, explaining how people responded when he said he wanted to go to medical school.

*“Others said would they perceive me as, you know... Big fat Doctor, and if I’m giving them advice about healthy eating, wouldn’t they just think... you can’t swing that sword at me when you can barely control your own eating.”* (Steve)

Our participants did not, however, accept these stereotypes. For example, Steve explained that *“I’ve never associated myself as being… You know, inherently unhealthy.”*

Despite this shared lived experience, our participants’ sense making around these experiences varied. Hannah, in particular, had given this a lot of thought. She had read widely around this topic as part of her own reflection and sense-making process, resulting in her acknowledging the influences of wider societal structures on ‘obesity’ and the way higher-weight people are viewed.

*“And in the same way, yeah sure each fat person is feeding themselves, but we’ve got… an obesity epidemic. That means there are structural problems in our society in how we live and how we exist that is creating this.”* (Hannah)

Hannah seemed to evidence a more sociological criticality in identifying the cultural norms that deem it appropriate to treat higher-weight people poorly.

*“People are rude to fat people, they just are… Fat phobia and cruelty towards fat people seems to be something that we somehow think is acceptable.”* (Hannah)

In contrast to Hannah, Steve’s and Carmen’s recollections painted a picture of two people, although sensing wider influences, not quite reaching out to critically unveil them, being somewhat passively carried through life. In Carmen’s case, this took the form of descriptive accounts of negative interactions with specific others, but without demonstrating explicit awareness of wider societal influences on her experiences. Steve, while aware that other people did not treat higher-weight people well, protected himself through conceptualising potential reactions to his visible “deviance” as a normal part of the human experience.

*“And now I think that that’s part of the human condition… Everybody feels a bit isolated from everyone else… You feel a bit more so if you’ve got something that makes you different to everybody.”* (Steve)

His sense-making process generated an illusion of control within a perceived helpless situation, achieved through normalisation. This sense of autonomy provided a shield from harm. By proactively deciding his own outlook on life, irrespective of specific experiences, he was in control, not the world.

*“I’ve quite enjoyed being different in many respects, and I certainly try and think differently to everyone else… I think it’s something that I’ve tried to embrace a little bit.”* (Steve)

Hannah, through her own criticality, may have pinpointed a reason for these differences in conscious awareness of the sociocultural origins of these negative personal experiences and unwillingness to challenge the status quo: *“I think all of us have internalised fat phobia just from the world that we live in.”*

### GET 2: Medicalisation and education – How can I reprimand people for their weight?

#### Thematic mapping.

This theme narrows in to consider how experiences of medical education more specifically impacted participants, their thoughts, and their beliefs.

Entering medical school was not a positive experience. Where their prior lives had allowed them to detach from the societal discourse around weight to some extent, their medical studies did not.

*“I always saw myself as being reasonably healthy... Now it’s all my own self-doubt... a lot of my own perceptions of myself are quite negative, in part because... I’m fat now... It’s difficult to… to look nice, and it’s difficult to present this image that you’d like to as, as a doctor... I don’t think that the key demographic necessarily of medical school are people that are larger... they all seem to be quite normal size.”* (Steve)

“*At school, it didn’t affect me that much, like not as when I started med school because... like being aware of my weight and being conscious about it really affected me. … [Before attending medical school interviews] I wasn’t like aware of [how people judge weight].”* (Carmen)

The medical discipline was a force that drove this discourse – so they had entered an environment where ‘overweight’ and ‘obese’ were demonised and taught, without exception, as states of being to be avoided. Didactic teaching centred around pathophysiology and negative health outcomes, creating a sense of fear and urgency. As Carmen explained, *“it’s something that scares you”* to attend as a higher-weight student. Hannah echoed this. Digging deeper, Hannah felt the medical construct of obesity itself provided flawed foundations for their education to rest upon.

*“If we thought of obesity as a normal variant of body condition… we could talk then about obesity in a much more productive way.”* (Hannah)

As equality and diversity were being increasingly celebrated in contemporary culture, Hannah and Steve were aware that a critical movement had increased awareness of the social harms stemming from the overmedicalisation of difference. Despite this, our participants’ experiences of medical education belied this purported shift in philosophy. Hannah, for example, remarked that medical schools are years behind in their understanding of, and approach to, true acceptance of higher-weight people.

*“I think the very concept, the very idea that maybe someone in a bigger body might be healthy is not something that my medical school, or probably any medical school, is ready to accept. It feels like we are still a very long way away from that concept being accepted.”* (Hannah)

This blinkered approach to weight within medicine was also evident in participants’ experiences outside of the classroom. For example, various experiences with medical professionals on clinical placements evidenced wider issues. In particular, witnessing explicit anti-fat bias and the overt dehumanisation of higher-weight people seemed commonplace.

*“There was always a discussion about the biggest patient they ever had, [who] was this enormous person who they couldn’t fit in X, Y, and Z. So, yeah... there was definitely a kinda freak show element at the end of every discussion of it… I think the humanity just gets lost over and over again”* (Hannah)

Hannah heatedly discussed the cultural issues in the wider profession at length, grounded in her own experiential learning on clinical placements. These experiences led her to believe there was a hatred of higher-weight people within the profession, granting a license to treat them as lesser beings or to humiliate them. She felt this led to not only poor care, but to increased mortality, through diagnostic overshadowing and confirmation bias, whereby new problems seemed to be attributed to their higher-weight status.

*“It’s that… sort of sneering, like, side of it and I think… like, how do we treat fat patients, how do we talk to people with fat bodies.”* (Hannah)

*“It kinda makes me so angry… fat phobia is literally killing patients.*” (Hannah).

Digging deeper into her own sense-making, Hannah felt that medical professionals had to “bully” higher-weight patients in order to be part of the healthcare professionals’ “club” – to fit in with medical culture. Her experiences had taught her that dehumanisation and reprimand were important aspects of patient education. She found this highly counterintuitive, however, shifting a sense of “blame” onto patients, grounded in a widespread lack of understanding of social factors.

*“We don’t have any solutions to it (obesity) and yet you know we continue to bully patients by saying it’s your obesity that has caused X, Y, and Z.”* (Hannah)

*“There are just as many obese nurses as there are obese members of the public in general… Whereas doctors not so much and basically the reason is because they are wealthier… If you tried to eat the ‘eat well plate’ on the minimum wage, you’d spend 2/3rds of your wage on that food.”* (Hannah)

While higher-weight status is undoubtedly linked with socioeconomic status, the mechanisms underlying this relationship are not straightforward [64,65]. Thus, Hannah, for all her awareness of the social construction of the modern “obesity epidemic,” here lapsed into more dominant narratives that higher-weight status is the result of poor choices, albeit dictated by economic necessity – indicating that she was not fully immune to the internalisation of this rhetoric.

Whilst Carmen was not as critically aware of the “bullying” behaviours that Hannah perceived as expected, she did feel a sense of inner conflict. She was aware that her medical studies had taught her it was her duty to stop people being ‘overweight’ or ‘obese’. This was introspectively troubling, given her own higher-weight status.

*“I think it’s the stigma… If you’re a med student or a doctor and… you tell a patient ‘oh you need, you need to lose weight’ and then you are overweight yourself.”* (Carmen)

Despite the perceived need to remind patients of their higher-weight status, which was shared by all participants, none had fully embraced an individual-blaming rhetoric. None felt weight was as simple as individual behaviours. Their specific reasoning differed, however, based on their own background experiences and beliefs.

*“Whether there’s a genetic component I don’t know…”* (Steve)

*“I think the other difficult thing is that what the medical establishment is hiding underneath all of this, is that they don’t have any answers for us.”* (Hannah)

*“[Obesity] doesn’t come at all from like overeating… It can happen from several reasons, literally.”* (Carmen)

Despite this dichotomy between beliefs and taught practices, none spoke out. Instead, all chose to remain silent on this matter. Steve shrugged off the issue, maintaining his positive outlook. Carmen internalised it as an issue with herself – she felt she was a hypocrite for not being thinner. Hannah, on the other hand, had ruminated over the possibility of challenging this culture. However, despite this consideration, her experiences in medical education had taught her that such outspokenness might be unwise. Discussing the hierarchical nature of medicine, she seemed helpless against the positions of power her teachers had attained over their careers. As such, she remained silent and, to some extent, questioned her own morality and beliefs.

*“It’s very difficult because these people who are giving us these lectures are often consultants… They have spent their entire careers working in an area and there is a part of me that feels like I want to respect their conclusions.”* (Hannah)

After deep reflection, Hannah did stand by her own beliefs, but felt it best to keep her head down, to do as was expected and to graduate from her medical studies. For example, while maintaining her critical insights internally, she intended to externally conform in order to pass her exams.

*“All of our exam questions are based on, erm, our lectures, so… if it’s in the lecture it’s how I need to answer in the exam.”* (Hannah)

### GET 3: One size does not fit all – How can I be a professional when I am like this?

#### Thematic mapping.

This theme now steps away from culture and digs deeper into the physical environments in which participants were studying medicine, alongside the impacts of these.

The aforementioned cultural issues aside, physical environments presented another stressor for our participants. They were existing in spaces never designed with them in mind. In Carmen’s case, the act of simply existing in the physical world as a higher-weight medical student made her self-conscious and filled her with a sense of shame for taking up other people’s space in educational or medical settings.

*“If you are a larger person, you’re more self-conscious about your movement… You need to be conscious about like the others around you… you just have it like being repeated in your mind… it’s much more upsetting.”* (Carmen)

Physical spaces at their medical school presented a variety of challenges, and even injuries. For example, Steve and Hannah experienced years of pain and anxiety centred around ill-suited seating in lecture theatres.

*“If you’re sitting through lectures for three hours and you’ve got something digging into you the whole time, like, that’s rubbish”* (Hannah)

*“I always feel like I’m gonna fall through one at some stage.”* (Steve)

Carmen was consistently finding that her medical education caused physical pain. She recalled a variety of settings in which she felt her weight caused such problems. For example: *“In the dissection lab… [We] stand for really long… I get back pain.”* She internalised this as a personal problem, feeling she was to blame because she was not trying hard enough to lose weight alongside her busy medical studies. In fact, this internalised ‘blame’ for environmental and situational mismatches at medical school had Carmen considering extreme measures to escape this pain – to find a way out of a harmful situation.

*“Since I started my [medical] schooling I have been more aware of my weight… I’m really considering… bariatric surgery… [I] think that if I had it, it will make my life easier.”* (Carmen)

Specific environmental stressors also extended beyond buildings and space. A particular fear playing on the minds of all three participants centred around clothing. Various educational and clinical settings required uniformity. For example, Steve and Carmen recalled specific experiences where they were unable to find scrubs in their size.

*“Despite what they said on the… the label… they were horrible… I think there’s nowhere to hide... Absolutely so terribly unflattering…The one size fits all really doesn’t.”* (Steve)

*“[I asked if they have] large sizes and then they told me, oh no, we don’t…”* (Carmen)

This was a real fear, as scrubs were mandatory attire in clinical settings during the COVID-19 pandemic. As such, they faced a difficult choice – comply or risk reprimand. On the one hand, they could have attempted to explain the situation to busy clinical staff who may or may not have understood. On the other, they could have tried to force themselves into inappropriately sized clothing. In Steve’s case, he chose the latter. This led him to experience a sense of both humiliation and shame on clinical placement.

*“So, I literally just had to pull up the scrubs and then… tear them [a] little bit to sit down. That was kind of the worst experience for me… It felt like I was interrupting with the flow of what needed to be done by being too big to fit in these fucking awful scrubs.”* (Steve)

Interestingly, more broadly, clothing held a deeper sense of meaning for both Steve and Hannah. In recalling their experiences, it became obvious that their size – and by extension their clothing – was inextricably linked with their sense of professionalism. Visibly higher-weight status and a perception of professional identity felt mutually exclusive. In Steve’s case, he felt that higher-weight status itself was an unpresentable way of being.

*“I will always have the perception that they (thinner peers) look better, that… they come across as somebody that’s more presentable.”* (Steve)

For Hannah, whilst the sentiment was shared, she did not believe that higher-weight status was inherently unpresentable or unprofessional. Instead, she considered external factors to which she could attribute this perception. She situated the problem in a lack of consideration of higher-weight bodies when clothes are designed.

*“As a medical student you have to look smart… Finding smart clothes for bigger women is not the easiest thing in the world...”* (Hannah)

### GET 4: Peers, patients, and professionals – It’s normal to talk about people like that, right?

#### Thematic mapping.

This theme now moves on to explore the interactions between people within the aforementioned environments.

Entering medical school was an awakening. Our participants were suddenly immersed in a new culture with its own language and practices. Whilst learning where they fit within this culture, they were exposed to a cornucopia of new people. First and foremost, they were assimilated into the medical school environment itself – with university staff and a cohort of medical student peers. For the most part, their experiences of direct interaction with others in university settings were positive. For example, Carmen explained that *“I think in terms of staff and students.... Until now they’re really like respectful, and they don’t mention anything about [my weight].”* Despite this surface level acceptance among the cohort, however, Hannah’s experiences had led her to believe that her peers had been judging her weight silently.

*“So, this is interesting… No one mentioned my weight when I was fat… But since I’ve lost weight, people have mentioned it.”* (Hannah)

More overt problems arose when our participants were not the focus of conversation themselves. Being a “fly on the wall” allowed glimpses of insight into the perceived mindsets of peers. Hannah, for example, passionately recalled an experience where she witnessed her peers discussing a deceased higher-weight person in disgust. This harsh reality both upset and angered Hannah. Whilst she was angered on behalf of the person, her emotions went deeper. Hannah’s reaction felt visceral. She was experiencing a glimpse into her own future, seen through the eyes of her peers.

*“Seeing the disgust of medical students as they were dissecting his body and, you know, complaining about how long it took to kinda clean away the fat… It was interesting to me because I looked across and I thought, you know, if I donated my body, would they be saying the same thing about me… There was a part of me that kinda wanted to bash the other students’ heads together and be like can you please stop complaining, this man has given you the most incredible gift.”* (Hannah)

Leaving the university environment for clinical placements, or for concurrent clinical work, was like entering the real world, where others no longer held back disdain. Our participants were exposed to the high-pressured healthcare sector in all its rawness, without the shield of a controlled educational bubble. Steve, for example, recalled how one patient had called him “*a terrible fat person.*” In keeping with Steve’s wider mindset, he dismissed this event as something that was a normal part of clinical life, feeling he was not being ‘singled out’. Reflecting on this, he explained “*that’s more [reflective of] their own illness*” – compassionately rationalising the situation and maintaining his positive outlook.

All our participants recalled negative experiences interacting with healthcare professionals. In some cases, this was more overt than others. The underhanded meaning, however, was apparent to all. In Steve’s case, he recalled how a consultant had consistently dropped hints about his weight in public until, eventually, they decided to tell him outright to lose weight, apparently feeling this was in his best interest.

“*He did kind of make anecdotal remarks about this old friend that he had that was too fat… constantly… which I knew was subtly, very subtly directed towards me…”* (Steve)

*“[This consultant] had also been a little less subtle... saying... look, come on, you need to lose a bit of weight.”* (Steve)

Once again, Steve rationalised this by explaining that the consultant was “*going through his own sort of massive health kick*.” When making sense of the experience, he did feel the behaviour was unkind, but he chose not to speak out. At face value, his reasoning was that the consultant was “*generally well liked.*” There was, however, also an underlying hierarchical component, where Steve referred to the person as “*a fairly senior consultant*” more than once. After discussing several similar experiences, it became clear that Steve had come to view this as normal behaviour in clinical settings. After all, “*I do tend to get the look up and down*.” (Steve)

*“Obviously in that moment, when the consultant is doing their eyes up and down, you don’t necessarily say anything...”* (Steve)

Carmen’s experiences were not dissimilar. However, where Steve was targeted directly, Carmen was more passively othered. For example, clinical staff would make negative observations or comments about higher-weight people and then panic about Carmen’s presence – “*I hope you don’t get offended by this*.” When asked how one example of this made her feel, Carmen stated she “*didn’t like*” it. It was clear from the conversation that there was more to this feeling, but she was not yet ready to emotionally process these interactions.

*“When she mentioned being overweight and like she just like related the incident to me, you know.”* (Carmen)

Hannah’s experiences were interestingly unique. Where the others had recalled a host of negative things people said or did to them, Hannah was more focused on what was *not* said. As medical students, our participants were privy to a cacophony of conversations about patients. Hannah had reflected on these extensively to better understand her experiences. Whilst she was still medically classed as ‘obese’, she had lost weight during her medical studies. Along the way, she had noticed a key difference in the content and depth of these conversations behind closed doors. When making sense of this, she realised that her weight loss seemed to have given doctors the free rein to now speak as they truly felt.

*“I feel like, in placement, there’s been a few times where… I think they’ve said different things in front of me because I’ve lost the weight... So, in previous consultations… when obesity was discussed… I think they would kinda skirt around it… Whereas now… they’ll just be very open about saying how unhealthy someone is or they perceive someone to be because of their size.”* (Hannah)

Providing another perspective, Carmen had also been in the position of seeing a doctor herself recently. Whilst she was also a medical student – learning to join the club – this visit concerned her own health. Given her medical training, she was able to analyse the consultation through a new lens, seeing patterns she may not have noticed prior to attending medical school. Where she may previously have dismissed seemingly irrelevant remarks about her weight as being somehow linked to her presentation in a way she did not understand, she was no longer able to rationalise this. In this instance, from the other side of the consultation, she witnessed first-hand the position of power a doctor can hold. Being on the receiving end of this hurt. In her own words, “*I got so mad*.”

“*I went to the GP for like something and then… immediately she started to mention my weight… she told me ‘how about I refer you to a dietitian… because… I clearly see your weight is an issue’… I didn’t consent to be referred… Then then they told me, ‘are you sure you don’t want to go to the dietitian? Because clearly you have a high BMI’*.” (Carmen)

### GET 5: Emotional impacts and the sense of self – How can I become a doctor looking like this?

#### Thematic mapping.

This theme now pulls all of the prior themes together in exploring the impacts of the aforementioned experiences on participants.

Despite varying degrees of awareness or acceptance, all three participants appeared to have internalised their experiences. This internalisation seemed to impact their self-concept, their general wellbeing, and their subsequent behaviours. Carmen, for example, felt like an imposter and a hypocrite – a fraud, even – due to “*the stigma*” surrounding her weight. How could she tell patients to lose weight looking the way she did?

*“I don’t want to say ashamed, but… I’m not accepting myself. I’m not accepting… my body image.”* (Carmen)

This seemed to reflect the psychologically wounding impact of Carmen’s experiences. She felt guilty about allowing herself to be a higher-weight person, attributing this as an internal fault – a flaw in her character – as this felt at odds with her medical student identity. She believed that, if she did not lose weight, future patients would consider her “*lazy*” because “*people always associate obesity with laziness*.” This mindset was consciously upsetting to her, but each negative experience further reinforced it.

*“So, like, it makes me really… upset, like, you judge a person based on their body type rather than the personality...”* (Carmen)

Steve, on the other hand, was ostensibly hidden safely behind a tough façade. He decided to view the world through rose-tinted glasses, maintaining his positive outlook on life. In particular, he made substantial effort to rationalise others’ behaviour in line with this, working hard to find alternative explanations or framings. In some ways, this did preserve his wellbeing. As he put it: “*It’s never affected the way I think or… how I look at myself or feel.*” However, this was only surface deep. Digging a little deeper, it became clear that, by not processing his experiences, he was shielding himself from their potential impacts. However, much like Carmen, when he was truly honest with himself, he too felt like an imposter. He felt unworthy to join the medical “club” as a higher-weight person.

*“I suppose the biggest thing that’s ever really affected is my own self… confidence in my own perception of whether I’m worthy to be a medical student or a doctor.”* (Steve)

Recalling her story, Hannah was the most open about her emotional journey. More than anything, she felt “*ashamed*” and “*frustrated*” about what she had to experience as a higher-weight medical student. These experiences led her to become distrusting of those around her in the medical world. In particular, she believed her medical student peers considered her unfit to become a doctor due to her weight. She worried they were thinking this behind her back. This appeared to be a learned behaviour, shielding herself from the further harm that could accompany any unexpected negative interactions. Assuming the worst in this way had become a survival mechanism in an environment of perceived hostility.

*“[I worry that] they’re looking at me and going ‘why do you want to be a doctor if you’re unhealthy like this?’”* (Hannah)

Interestingly, Hannah’s weight was so intertwined with her identity that she was able to reflect openly about this. Being a higher-weight person was a core part of her being and, as such, deeply impacted her sense of self and feelings of belonging. So much so, in fact, that losing some weight had negative impacts for her. Who was she now? What was left?

*“I’ve lived my whole life as a fat person and, you know, as somebody who was restricting, restricting, restricting in order to try and not be a fat person… So, now that I’ve lost a certain amount of weight, it’s really difficult… [In my] identity, like, I’m still obese.”* (Hannah)

### GET 6: Future worries and wonderings

#### Thematic mapping.

This theme now draws our analysis to a close through considering participants’ views regarding their futures within the medical profession as a result of their reported experiences.

Not yet being qualified doctors, our participants naturally worried about their futures. The aforementioned wounds in their self-concept and sense of worth, however, amplified these worries. Recollections of past experiences were often framed within these future fears, reinforcing feelings of being imposters or of being ill-suited to the profession. There was a sense of impending doom within this, as though this may become a self-fulfilling prophecy. Steve and Carmen, for example, were particularly worried about the impacts of their weight on their ability to function as doctors. Steve felt anxious that he would not be able to contribute to acute patient care efficiently due to his weight.

*“I think it’s my biggest concern, and if there’s a trauma call going off that I’ve got to attend and you know, am I feasibly going to be able to run down the corridor to attend.”* (Steve)

Carmen echoed this feeling. There was a sense of being left behind evident in her worries. She longed for people to recognise the challenges faced by higher-weight individuals and to introduce adaptations into the medical workplace. She was not, however, confident that this was likely to happen in the real world of medical practice. If she could not keep up, this would be her fault, and no one would wait for her.

*“I’m really worried that I might – I may get tired… or, I cannot keep up with the rest…”* (Carmen)

Hannah, however, took a different view. It was clear that she hoped to overcome such fears through taking control of her own narrative and focusing on the longer-term goal. She was determined to survive her experience in medical school. This drive stemmed from a moral imperative to improve things for higher-weight patients from within the system. If she feigned compliance (see GET 2) enough to complete her education and training, she could then use her respected position to advocate for change. This was in keeping with her previously mentioned perception of the medical hierarchy and its impacts on prevailing discourse (see GET 2).

*“…just sort of talking to them about [the fact] that I understand, you know, what it’s like to live in a fat body and how difficult it is to manage whatever conditions they are going through as well as just trying to exist in the world in a fat body… People, I suppose, who are bigger, sometimes don’t have the best experience with medical professionals.”* (Hannah)

## Discussion

Following on from previous work grounded in our own experiences [44], this is the first in-depth study of the experiences of higher-weight medical students in a UK context. Our results highlighted a variety of issues faced by our participants. These included an awareness of societal dislike of higher-weight people, challenges working and learning within the medical system as higher-weight people, and a negative emotional burden associated with this endeavour. Despite these challenges, Hannah acknowledged the importance of retaining higher-weight people in the medical workforce to support and advocate for higher-weight patients. This work builds on the previous literature, which has documented issues with both implicit and explicit anti-fat bias in the medical field [46,47].

Hannah’s discussions around societal issues were interesting. Higher-weight status is both born of and demonised by modern society in general. Throughout our study, however, it also became clear that participants perceived higher-weight status as both an identity and a role, with its own associated preconceptions and expectations relating to both behaviour and ability – one should be lazy and one should overeat, for example. As such, despite weight’s variable nature across one’s life course, higher-weight status seemed to be associated with a subculture of its own. This was further supported by our participants’ experiences of marginalisation and othering, alongside the wider societal scapegoating of higher-weight people as a distinct group – driven by the moral panic surrounding obesity, leading to suggestions of both self-infliction and economic burden [12,13]. In Hannah’s case, this likely fuelled the reported conflict of self-identity when she lost weight. The concept of ethnocentrism explains how cultural groups tend to view other groups as lesser than their own – viewing their practices and beliefs through the familiar cultural lens, and thus interpreting them as alien or unusual – supporting in-group favouritism [66]. This can leave minority groups (the out-groups) open to cultural imperialism, whereby their own culture (or subculture) is overwhelmed in favour of the values and practices of the majority [67]. In that sense, non-conformity may expose its members to marginalisation and ill-treatment through a perceived deviance. Given the visible nature of higher-weight status, it would be impossible to feign such conformity in the hope of improved treatment from others. This hyper(in)visibility [68] may, in part, explain some of our participant’s fears of ill-treatment or judgement from their colleagues.

There is a prevailing discourse, including within a UK context, that ‘obesity’ is self-inflicted, with higher-weight people held personally responsible for their ostensibly immoral state of being [69]. This may be driven by overmedicalisation, rather than recognition of diversity and the correlation between weight and the social determinants of health [64]. In the context of our study, it seemed that this medicalised view had been somewhat internalised by our participants, reinforced by their medical studies, and it was clear that this influenced their wellbeing and day-to-day life choices. For example, Steve felt the need to actively defy this preconception, whereas Carmen displayed some helpless attributes regarding her feelings of self-worth and her self-perceived abilities. Regardless of their specific views, all three participants experienced a negative impact on their self-worth and/or wellbeing through starting their medical studies. Steve reported no impact of his weight on his wider life prior to becoming a medical student. However, following some time at medical school he found himself feeling inferior due to this – considering “*whether I’m worthy to be a medical student or a doctor*”. This was echoed by Hannah through her worries that other medical students and doctors might be silently judging her based on her size, seemingly confirmed by the change in discussions when she lost weight. In Carmen’s case, this was evident through her newfound worries that her role as a doctor as she perceived it – to identify patients as being too overweight and having to “tell” them to lose weight to be healthy – was at odds with her own appearance. This made Carmen feel like a hypocrite, leading to inner conflict. However, it would be remiss of us not to also consider the risk of moral injury here. Moral injury refers to a form of “trauma characterized by guilt, existential crisis, and loss of trust that may develop following a perceived moral violation” [70]. In essence, this may occur when someone undertakes tasks that betray their morality – something they know to be wrong per their own moral compass [71]. Its application here is clear. As Lomax-Sawyers, writing as both an insider as a medical student, and outsider as a higher-weight person, previously explained: “bludgeoning fat people with a suggestion we should be less fat every time you see us actually doesn’t make us less fat. It makes us avoid going to see a doctor when we need one. But most importantly, it makes us feel awful, and contributes to the already crushing stigma we experience living in our bodies every day” [72]. Whilst such practices signal weight stigma and healthcare avoidance in higher-weight patients [24], it is also clear that training our higher-weight students, as insiders to both worlds, in such practices may risk inflicting moral injury.

Finally, we must not overlook considerations around intersectionality. Whilst Carmen and Hannah reported notable negative mental health challenges relating to their experiences, Steve did not. This is in keeping with the wider literature on weight stigma, which has found it to be an intersectional issue in the way it is internalised [73]. For example, studies show that women experience higher rates of weight stigma [74], as do racial and ethnic minorities and individuals of lower socioeconomic status [65]. Individuals belonging to multiple marginalised groups, report experiencing more frequent stigma, and experience worse health outcomes than singly stigmatised (and non-stigmatised) individuals [75,76]. In the present study, it could be argued that Steve’s positive outlook suggests that the other participants are simply “too sensitive” and that they simply need to “toughen up” in this highly competitive environment. Indeed, evidence from a study of weight-related microaggressions in US medical students found that, compared with normative-weight students, higher-weight students reported *observing* more weight stigma directed toward other higher-weight students or patients, which might support such a contention [21]. However, research suggests that members of stigmatised groups are not hypersensitive to microaggressions, but simply experience them more frequently [77]. Furthermore, while individual responses to a particular demonstration of stigma may vary, microaggressions by their nature represent a contextual signal underpinned by the unequal, and lesser, status of some groups, and their expression serves to perpetuate that inequality [78,79]. And while individual instances of anti-fat bias in everyday life may seem relatively minor, it is the cumulative nature of these signals that create a hostile environment for members of a marginalised group, and it is this chronicity, rather than acute events, that is linked with poorer long-term health outcomes [80]. Importantly, neither the source (interpersonal versus environmental), the target (direct or observed), or the intent (malicious versus well-meaning), is a major moderator of resultant harm [81]. For example, a recent study identified that benevolent weightism – sometimes (although not always) well-intentioned comments about weight-loss – is just as harmful as overt negative treatment, and was linked with greater perceived stress, trauma symptoms, self-reported health, and self-restriction of life choices (e.g., in education and employment), and that this process operated in part through reducing self-worth [81]. As weight is not a protected category (in any country), no data are available on medical school application, acceptance, or progression by weight status. It is clear, as noted by Hannah, that higher-weight medical students are an under-represented group when considered alongside rates of higher-weight status in the general population. It would be of interest to discover whether this is due to admissions bias or to self-selection out of the applicant pool, although it seems likely that both play a role [21]. Interestingly, in a study of everyday fat microaggressions, “clothing exclusion” emerged as a prominent form of weight-related microaggressions, representing an everyday indignity that may seem a minor inconvenience to those unafflicted with this issue, but “communicates to everyone that fat bodies are not valid, legitimate, or deserving of clothing at their larger size. That is, clothing does not exist for bodies that should not exist” [81]. This supports our participants’ experiences that inaccessibility of suitable clothing was a constant reminder that they did not belong and resulted in them wondering if this was indeed true. Even Steve, who claimed to be unaffected by the weight bias he experienced, when questioned, admitted to impostor syndrome and concerns about his future in medicine.

Considering intersectionality, alongside all of the aforementioned issues, in the context of allostatic load, these added role conflicts (medical student vs higher-weight person), prejudicial interactions, and potential moral injuries may negatively impact on their own health down the line – both mental and physical [82,83]. This could be devastating for our higher-weight students. As medical educators, we must therefore ask ourselves: what is happening to induce such worries and feelings of inferiority in our higher-weight students? What aspects of the hidden or indeed explicit curriculum might be acting to impact them so negatively? And how much of themselves, their wellbeing, and their later health should our students be made to sacrifice to gain entry into our profession? Whilst influencing the discourse and attitudes in the wider medical profession (to which we are exposing our students) is a longer-term goal, we can and should be seeking to understand and improve matters during their medical studies. Further research is needed to explore this area in depth.

### Considering the future

In line with the GMC’s Welcomed and Valued guidance [32], and much like with other marginalised groups, higher-weight doctors are more likely to intuitively understand the systemic issues faced by higher-weight patients and, therefore, have the ability to empathise and deliver improved healthcare. This supports Hannah’s position on the potential benefits to higher-weight patients of having higher-weight medical professionals. It is therefore key to support inclusive practices for higher-weight students and trainees within medical education. We make the following recommendations for medical school curricula and institutional changes, some of which can be implemented relatively easily whilst some will involve a longer-term commitment to inclusivity and diversity within institutions.

1. Medical schools should review and update their weight-related teaching. However well-intentioned, the focus on weight and weight loss grounded in our medical training may in fact be driving increased patient morbidity and mortality [44]. As outlined in the Introduction, an extensive and growing evidence base now challenges the weight-centric paradigm and suggests a weight-neutral or weight-inclusive approach to healthcare will deliver improved outcomes. Institutions should draw on resources and training available from the increasing number of organisations with specific expertise who are advocating for change in this area. For example, ‘Medical Students for Size Inclusivity’ [84], the ‘Association for Weight and Size Inclusive Medicine’ [85], and the ‘Association for Size Diversity and Health’ [86]. Students should be made aware of the existence of these organisations.
2. Medical schools should consider weight stigma as part of their current efforts to decolonise medical curricula across the UK, including body size as part of the wider social justice-informed approach to medical education. Tackling these issues early with education at medical school may lead to an improved understanding and an altered discourse in the medical workforce, leading to improved experiences for both staff and patients.
3. Medical schools should review their facilities, material resources, and dress code policies to ensure these are inclusive for higher-weight students – for example, through the provision of larger scrubs and lab coats where these are required. The wellbeing of higher-weight (and other) students should also be considered within aspects of the curriculum requiring prolonged static standing, where seats could be provided as part of universal design. Finally, where facilities are due to be updated, consideration should be given to the width of available seats, load supported, availability and location of arm rests, and widths of aisles – to reduce both physical pain and an undue sense of shame. This is in keeping with recommendations in the wider higher education literature [87].
4. Higher-weight medical students should be actively included within the above processes. Their lived experience and insight should be valued, and they should be empowered to help shape the future of medical education.

### Strengths, limitations, and reflexivity

Through a constructionist lens, strengths and limitations can be highly contextual – socially, politically, and historically. What one reader may applaud as a key strength, another may consider a limitation, grounded in their own background, milieux, and beliefs. One need only consider the ontological and epistemological disparities between positivism and constructionism, for example, to evidence this. Accordingly, whilst we do not set out to debate matters of philosophy here specifically, we approach our strengths and limitations in an accordingly reflexive and inter-twined manner. For example, despite our social constructionist beliefs, we have ensured to include all aspects of the Standards for Reporting Qualitative Research (SRQR) guidance [88] in reporting this study – a requirement of the journal. While a constructionist may consider this a constraint, framing this in an evidence-base on the “tyranny of method” [89] that betrays the more creative and artistic groundings of qualitative research [90], a critical realist or positivist reader may consider this a strength.

As the first study, to our knowledge, to explore the experiences of higher-weight medical students in a UK setting, this work provides a unique and useful insight into their social world. The small, homogeneous sample from a single UK medical school was ideal for a phenomenological study [56]. Whilst the results are not generalisable – an important limitation if viewed through a positivist lens – they do not seek to be. Instead, grounded in social constructionist views, our study sought to explore and interpret the experiences of our participants – from their unique perspectives – to provide deeper meaning and understanding. The findings may be transferable to other settings, and such considerations are grounded in the minds of our readers. For those seeking assessment of generalisability, our findings could open the door to future work to assess this from a more positivist perspective.

Given the specific nature of our research focus, our findings may also benefit from advancing our emotional understanding of this experience, embodying an “epistemology of emotion” [91]. This is in keeping with the phenomenological aim to also advance knowledge non-cognitively [92]. On the other hand, considering *cognitive* knowledge, and drawing on the concept of C. Wright Mills’ “sociological imagination” [93], we have explored our participants’ personal troubles and, through a hermeneutic cycle [61], interpreted these in the context of potential public issues. SS and LH’s insider status was key to this process and, within the interpretive phenomenological approach, was an important strength. Furthermore, whilst some may also consider the insider positioning and activist stance of our research team a strength, others may also consider this a weakness.

SS and LH’s co-construction of our results is also worth reflection. Within our chosen reporting guidelines, consideration of such triangulation and validation of “trustworthiness” is key [88]. Under a constructionist approach, however, this is not strictly necessary – reflecting the inherent subjectivity of interpretive research approaches [57]. Reflexively speaking, we opted to triangulate and co-construct in this way for a couple of reasons. Firstly, this approach incorporated the insider understanding of both SS and LH. Whilst both are insiders to the world of medical education and being higher weight, SS and LH are distinct beings in their lived experience and their intersectionality. As such, we felt this approach offered a richer depth to our interpretive analysis. Secondly, this approach facilitated professional development within our research team, which is an important aspect of any researcher’s journey. Whilst SS is well experienced with IPA, LH came to this project with minimal experience of this method. Through her immersion in the analysis alongside SS, this approach embodied an apprenticeship-type approach to her own academic development.

## Conclusions

Our study explored the experiences of higher-weight medical students in a UK setting. This provided a window into their world. Participants reported logistic and environmental issues, such as lack of provision of larger uniform sizes or inaccessible lecture room seating. They also reported negative experiences with peers, teachers, and the general public in relation to their size. Despite this, higher-weight doctors were felt to be important to advocate for higher-weight patients. Negative experiences seemed to stem from wider sociocultural issues and a widely tolerated clinical culture. Higher-weight status comes with its own subculture, and thus ethnocentrism and cultural imperialism may be driving the dominant discourse, leading to stigma and fear of ill-treatment. Though an allostatic load lens this may risk negatively impact the health of our higher-weight medical students. To begin tackling these issues, we argue that medical schools should consider incorporating weight stigma into their curricula.
